# Body Representation in Patients with Severe Spinal Cord Injury: A Pilot Study on the Promising Role of Powered Exoskeleton for Gait Training

**DOI:** 10.3390/jpm12040619

**Published:** 2022-04-11

**Authors:** Maria Grazia Maggio, Antonino Naro, Rosaria De Luca, Desiree Latella, Tina Balletta, Lory Caccamo, Giovanni Pioggia, Daniele Bruschetta, Rocco Salvatore Calabrò

**Affiliations:** 1 Department of Biomedical and Biotechnological Science, University of Catania, 95123 Catania, Italy; mariagraziamay@gmail.com; 2 AOU Policlinico “G. Martino”, 98125 Messina, Italy; g.naro11@alice.it (A.N.); dbruschetta@unime.it (D.B.); 3 IRCCS Centro Neurolesi “Bonino Pulejo”, 98121 Messina, Italy; rosaria.deluca@irccsme.it (R.D.L.); desiree.latella@irccsme.it (D.L.); tina.balletta@irccsme.it (T.B.); 4 Neuropsychology Unit, University of Padua, 35121 Padua, Italy; lorycaccamo@gmail.com; 5 Institute for Biomedical Research and Innovation, National Research Council of Italy (IRIB-CNR), 98164 Messina, Italy; giovanni.pioggia@irib.cnr.it

**Keywords:** body representation, rehabilitative devices, robotic rehabilitation, spinal cord injury

## Abstract

Patients with spinal cord injury (SCI) complain of changes in body representation, potentially leading to negative physical and psychological consequences. The purpose of our study is to evaluate the effects of robotic training with the Ekso-GT on body representation (BR) and on the quality of life in patients with SCI. The trial was designed as a pilot, assessor-blinded study. Forty-two inpatients with a diagnosis of SCI, classified as either American Spinal Cord Injury Association Impairment Scale (AIS), were enrolled in this study and randomized into either a control (CG: *n* = 21) or an experimental (EG: *n* = 21) group. Patients in the EG received rehabilitation training with the Ekso-GT device, whereas the CG patients were trained with conventional physical therapy (CPT), which consisted of physical and occupational therapy and psychological support. We considered as a primary outcome the modified Body Uneasiness Test (MBUT), focusing on three specific subscales on the patient’s perception of BR, i.e., the Global Severity Index (MBUT-GSI), which is an indicator of body suffering; the Positive Symptom Distress Index (MBUT-PSDI) that expresses an individual’s psychological distress; and the Lower Limb MBUT (MBUT-LL), which indicates the subject’s perception of their thighs/legs. The Short-Form-12 Health Status Questionnaire (SF12) and the Beck’s Depression Inventory (BDI) were used as secondary outcomes to evaluate the effect of the training on the quality of life and the psychological status. Non-parametric statistical analysis showed that the effect of the two treatments was significantly different on MBUT (BR), SF-12 (quality of life), and, partially, BDI (mood). Particularly, patients belonging to the EG achieved a major improvement in nearly all test scores compared to those in the CG. Our data suggest that the Ekso-GT training could be helpful in achieving positive changes in BR in patients with chronic SCI, especially in reducing psychological distress (PSDI) and thigh/leg perception (MBUT-LL) with an overall improvement in quality of life (SF-12).

## 1. Introduction

Spinal cord injury (SCI) is damage that affects a portion of the spinal cord or the nerves of the cauda equina, resulting in a permanent or temporary loss of motor, sensory, and autonomic function [1]. SCI has an estimated prevalence of 54 cases per 1 million people and a hospital mortality rate of approximately 8% [2]. In Italy, the Italian Group for the Study of the Epidemiology of Myelolesions has found that the incidence of SCI is 2500 cases per year, with a prevalence of 60,000/70,000 cases per 60 million inhabitants [3]. SCI is commonly divided into complete or incomplete depending on the extent of the injury. In a complete lesion, the ability to send and receive messages from the brain to the body systems that control the senses, motor, and autonomic function below the injury level is impaired. The patient perceives no sensation below the injury site and cannot perform any voluntary movement. In an incomplete injury, the spinal cord can transmit some messages to/from the brain and the rest of the body. Therefore, some motor and sensory functions may be spared [1,2,3]. Depending on the location and severity of the damage, the symptoms and the long-term outcomes can vary considerably from pain and numbness to complete paralysis [4]. Complications of SCI include increased risk of infections, incontinence, vesicoureteral reflux, nephrolithiasis, and renal failure [3]. Due to the potential disabilities, SCI has a significant effect not only on activities of daily life but also on the social and psychological well-being and quality of life (QoL) [5,6]. Furthermore, SCI often affects young working-aged people, which leads to relevant socio-economic costs. Hospital rehabilitation requires time and high resources (multidisciplinary teams, dedicated equipment, spaces) in order to obtain optimal rehabilitation results [7,8]. Munce et al. found that inpatient rehabilitation costs for SCI patients attending a center in Ontario represented 58% of the overall cost of SCI expenses [9]. More recently, Gamblin et al. observed that rehabilitation costs accounted for nearly 90% of the facility’s total costs for SCI patients attending a U.S. Department of Physical Medicine and Rehabilitation from January 2011 to December 2017. These data may be due to the fact that hospital rehabilitation requires more resources for rehabilitation activities (i. e. physical therapy, recreational activities) than other healthcare facilities [10]. In Italy, the estimated direct costs for patients with SCI are on average around EUR 26,900 in the first year after the injury and around EUR 14,700 in the following years [11,12].

Patients with SCI may experience changes in their body representation (BR), which have serious, negative physical and psychological consequences. BR refers to perception, memory, and cognition related to the body, and it is updated continuously by the sensory inputs [13,14,15]. BR includes: (i) body image, a conscious representation of the body, including the functions and relationships of the body parts between them as well as with the outside, and (ii) body scheme, a plastic and dynamic representation of the spatial and biomechanical properties of the body [16]. The two concepts are divided only for research purposes, as they are actually part of the same component [1]. BR is constantly evolving and influences life and interpersonal relationships. The well-known brain neuroplasticity allows also for the reorganization of bodily perceptions based on external internal feedback [17,18,19]. BR includes the perceptions and attitudes of self-related to the body, such as thoughts, beliefs, feelings, and behaviors. These aspects of BR may affect psychosocial functioning and emotional stability [20]. In particular, negative BR experiences take place as the cumulative result of influences and specific events that trigger maladaptive processes [20]. It has been shown that BR may affect rehabilitation outcomes [21]. In fact, alteration to BR can lead to dissatisfaction with the body image, loss of self-esteem, reduction of general psychological well-being, QoL and mood of patients, as well as social isolation, further increasing the difficulty in moving in the surrounding environment [22,23]. Previous studies on BR in patients with SCI have focused on physical characteristics and physical activity to evaluate whether the self-perception in different physical conditions (paralysis of the limbs, difficulty in walking, increased physical effort) compared to the premorbid phase may have effects on the way patients perceive and interact with the surrounding environment, including how they relate to partners [22,23,24,25,26,27,28]. In fact, the physical and psychological components cannot be separated, so a significant change in one level obviously affects the other as well [22,23,24,25,26,27,28]. On the other hand, it has been suggested that body satisfaction improves over the years following SCI [29,30]. It is noteworthy that some authors found that patients tend to perceive the paretic parts of their own body as if they were active and dynamic even if paraplegic [31,32]. Although some studies have found that BR deterioration negatively affects the QoL [13,23,27,33], the relationship between BR and the emotional aspects about the adaptation to SCI has been poorly investigated [13,14,33]. As far as we know, there are only a few studies regarding the role of BR in SCI patients attending rehabilitation wards [15,34,35,36]. In their study, van Diemen et al. found that rehabilitation leads to an improvement in BR as well as in depression and anxiety, suggesting that rehabilitation should target interventions for BR changes [37]. In patients with SCI, rehabilitation mainly aims to reach the highest level of autonomy and functioning without paying due attention to the patient’s BR [38]. However, evidence shows a reorganization of the sensorial and motor systems after rehabilitative training in patients with SCI, with positive effects also on BR [38]. Although a wheelchair is the main mobility device for SCI patients with permanent or progressive disability [39], being able to reach the upright position and a normal gait is fundamental to SCI for both physical and psychological purposes, including self-esteem and BR. In particular, the standing position has beneficial effects on breathing (due to a reduction of pressure on internal organs and an improvement of lung volume and blood oxygenation) as well as on the circulatory system, including the prevention of lower limb edema and orthostatic hypotension [40]. Moreover, an improvement in both gastrointestinal functions [41] and bone mineral density [42] has been found as further evidence that normal standing and gait may improve non-motor functions in SCI. Concerning the psychological effects, it has been shown that standing has a positive psychosocial effect on the user since it increases QoL, happiness and independence, as well as self-esteem and the freedom to perform activities [43]. Conventional rehabilitation techniques, including motor relearning or proprioceptive neuromuscular facilitation, are effective in improving motor function, with repercussions on the BR [20,21,22,23,38,39]. However, conventional rehabilitation can require time to recover. 

**Figure 1:**
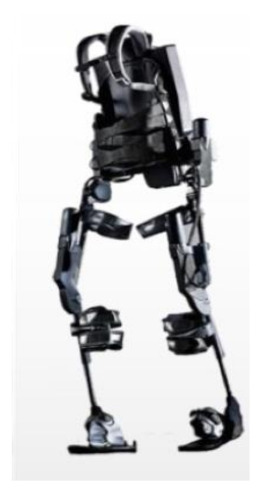
Shows the Ekso-GT™ device.

During the last years, new techniques for motor training have been developed, and these robotized devices are leading to promising results in terms of time reduction and less patient workforce [38]. In particular, exoskeletons, such as the Ekso Bionics^®^ Gait Trainer (Ekso-GT™) device (Richmond, CA, USA) (Figure 1), may help in reaching a nearly normal standing position and in walking [44,45,46,47]. 

Exoskeletons are particularly suitable for SCI people as they enable patients to achieve a better gait, walking, and even the possibility to climb stairs, thus making them more independent. However, these devices require specialized training and a significant degree of collaboration and effort by the patient concerning both the control method and the gait-initiation mode. Moreover, there are some specific manufacturing limitations concerning the user’s height and weight. Finally, the costs of exoskeletons are not negligible [48,49,50]. 

The rationale of adopting exoskeletons in SCI rehabilitation lies in their capability to provide patients with a free route of overground walking, which is a reliable motor practice, enabling the patient to get better control on his/her own body and surroundings owing to a big amount of sensorimotor and proprioceptive information. This can improve both motor outcomes and cognitive-emotional aspects, including BR, as well as social interaction and QoL. Such a gait practice fosters the improvement of proprioception, which in turn affects the emotional aspect of BR, as they are closely related [17]. It is known that BR is constantly evolving, as brain plasticity reorganizes a different perception of the body as result of internal and external feedback, influencing life and interpersonal relationships as demonstrated in a previous study involving patients with stroke [47]. 

Thus, the purpose of our study is to evaluate the effects of intense robotic training with the Ekso-GT on the BR and QoL of patients with SCI by using specific scales adapted to assess the patients’ perception of their BR. Our hypothesis is that robotic training could be useful for achieving positive changes in BR, which, in turn, may affect clinical outcomes.

## 2. Materials and Methods

### 2.1. Study Design and Population 

The trail was designed as a pilot randomized, assessor-blinded study carried out in accordance with the 1964 Helsinki Declaration and its subsequent amendments and approved by our Research Institute Ethics Committee (ID: IRCCS-ME 34/18). All participants signed an informed consent to enter the study. 

Forty-two inpatients with a diagnosis of SCI, attending the Robotic Neurorehabilitation Unit of the IRCCS Centro Neurolesi Bonino-Pulejo (Messina, Italy) between October 2018 and December 2019, were enrolled in this study. All patients were randomized (using block randomization with a block size of 2 × 2) into a control group (CG), which received traditional cognitive training, or an experimental group (EG), which underwent Ekso-GT™ training.

The patients were enrolled according to the following criteria: (i) age ≥ 18 years; (ii) diagnosis of SCI according to the AIS classification [51]; (iii) a stable SCI condition (i.e., at least 3 months after the injury); and (iv) ability to follow verbal instructions, with a Montreal Cognitive Assessment (MoCA) > 20.

Patients were excluded if they had severe bone disease, such as osteoporosis (T score < −2.5), severe pain, spasms, or spasticity (Modified Ashworth Scale > 3) despite treatment with specific drugs, and/or a history of concomitant psychiatric or medical illness (i.e., psychosis, epilepsy, colostomy, unsolved venous thrombosis, uncontrolled autonomic dysreflexia, skin irritations/lesions, and cardio-respiratory failure) potentially interfering with the training.

Both groups were provided with physical and occupational therapy (2 h/d), which was followed by an hour of gait training with conventional physical therapy (CPT) approach in the CG or the Ekso-GT™ device in the EG. This schedule was repeated five times a week for 8 weeks (from Monday to Friday) for a total of 40 training sessions. 

### 2.2. Conventional Physical Therapy (CPT)

CPT included physical and occupational therapy (2 h/d), aimed at improving muscle force and independence in the activity of daily living. In particular, it consisted of stretching exercises, endurance, standing, and assisted lower limb motor activities following the Bobath principles; muscle stretching and strengthening, balance training, postural stability control, sensory techniques, and functional daily activities occupational therapy; and functional electrical stimulation. The physiotherapist performed individual, face-to-face training sessions with each patient, focusing on improvement in flexibility, movement of the joints, strength of legs, verticalization, static balance on a tilt table, and overground walking during the one-hour gait training session. An hour a week of psychological support was also provided.

### 2.3. Ekso Training

Ekso-GT™ (Ekso™ Bionics, Richmond, CA, USA) is an exoskeleton for the lower limbs, equipped with electric motors to power the movement of the hip and knee joints. The device consists of an exoskeletal frame for the legs, passive joints of the ankle, a platform, and an electric motor. There is also a backpack that contains a computer, a battery, and a wired controller [47]. The exoskeleton is attached to the body by a series of straps fixed at a specific distance to ensure stability based on the patient’s weight [52,53]. In addition, the device incorporates a Smart-Assist software, which allows the physiotherapist to independently set the power of each leg to best suit the user.

Our patients’ training started with pre-gait exercises, including squatting and weight shifting. The physiotherapist was responsible for inserting the subject into the exoskeleton and ensuring his safety. Then, the first sessions were totally guided by the physiotherapist, preventing the user from falling thanks to the device backpack, which allows it to sustain the patient during walking. With the first step modality, the therapist used a push button controller to generate steps after appropriate weight shift. Gait parameters, such as step length and height, were changed by the therapists according to the patient’s clinical picture and improvement during training sessions. The Ekso GT™’s Variable Assist software allowed physical therapists to determine how much power could be provided to either side of the person’s body. This balanced the physical effort that the person exerted with the amount of help that he or she needed to achieve a more normalized gait.

With their improvement, the patients were able to walk in a progressively independent manner using the pro-step (i.e., the user stepped by moving the hips forward while twisting them) and pro-step plus modality (the steps were triggered by the patient’s load and the forward motion of the limb) and assistive devices, such as crutches or walkers. The Ekso-GT ™ was able to provide sound feedback to the patient. It helped to inform the patient whether he or she performed the load transfer correctly during the initiation of the step (with respect to the correctness of movement in the sagittal and frontal plane).

### 2.4. Outcome Measures

The Modified Body Uneasiness Test (MBUT) was the primary outcome. It focuses on the specific subscales that could be influenced by the rehabilitation training (Modified BUT—MBUT). As secondary outcomes, we considered the Short-Form-12 Health Status Questionnaire (SF12) and the Beck’s Depression Inventory (BDI) to assess the effect of the overground training on QoL and psychological status.

MBUT is a self-report of 34 items with a 6-point Likert scale (from 0 = never to 5 = always) that evaluates the discomfort related to one’s own BR through various factors [54]. To better adapt the scale to SCI patients, only three global scales that specifically refer to the patient’s perception of BR were taken under consideration: (1) Global Severity Index (GSI), which is the most sensitive indicator of the level of distress compared to the body; (2) Positive Symptom Distress Index (PSDI), which provides information on the average level of distress experienced by respondents; and (3) the Lower Limb item of MBUT, which indicates the subject’s perception of his/her own thighs/legs. In particular, this subscale evaluates the degree of distress felt by the patient towards his/her own lower limbs; low scores indicate poor acceptance and high discomfort concerning limbs’ shape and musculature. We believe that these indices are the most involved in the rehabilitation process followed by our patients since the scales refer directly to the perception of the body and not to other components, such as thinness or parts of the body not involved in the process (such as mustache or hairs).

The SF-12 is a quality of life questionnaire composed of 12 items that evaluate two different aspects of health: physical and mental health. The SF-12 is a scaled-down version of the SF-36, which itself evolved from the Medical Outcomes Study [55].

The BDI is a 21-question, multiple-choice, self-report inventory and is one of the most widely used psychometric tests to measure the severity of depression. BDI is a short questionnaire that can be done in 10–15 min; a total score greater than 14 indicates the presence of depression [56]. 

### 2.5. Statistical Analysis 

Data were analyzed using the SPSS 16.0 version, considering a *p* < 0.05 as statistically significant. Non-parametric statistical tools were used to analyze the data given that the outcome scales consist of ordinal data. Thus, we used the Wilcoxon and the Mann–Whitney test for within-group and between-group comparisons, respectively, corrected for multiple comparisons. The magnitude of the clinical changes (which provides valuable additional information regarding a test result that traditional null-hypothesis significance testing cannot) was assessed as effect sizes using Hedges’ standardized mean difference (g), and estimated as low > 0.2, moderate > 0.5, and large > 0.8 effects. 

The sample size was calculated in relation to the clinically significant changes in the primary outcome (MBUT) using a two-sided, two-sample *t*-test and estimated in 40 patients (20 per arm). This sample size is required to maintain a type I error rate of 0.05 and an 80% power to detect a significant pre–post between-group difference of 20% (39) (including a 10% dropout rate or loss to follow-up).

## 3. Results

No significant differences in age (*p* = 0.12), gender (*p* = 0.35), and education (*p* = 0.23) were found between EG and CG (Table 1). The range of the time post-injury was 4 to 9 months. No significant differences were found in the clinical assessment scores between the groups at baseline.

We found that both groups showed changes in each primary (MBUT-GSI, MBUT-PSDI, and MBUT-LL) and secondary outcome measures (SF12 and BDI). However, both the magnitude and the extent of the improvement depended on the type of treatment.

### 3.1. Primary Outcome

**Figure 2:**
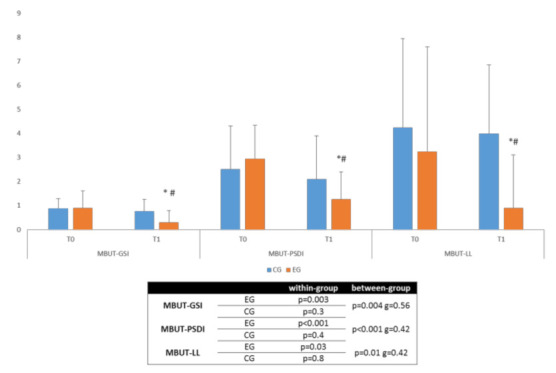
Primary outcome measures (median + interquartile range). * significant within-group differences, # significant between-group differences. Legend: EG, experimental group; CG, control group; T0, evaluation at baseline; T1, evaluation at the end of the protocol; MBUT, Modified Body Uneasiness Test; GSI, General Severity Index; PSDI, Positive Symptom Distress Index; LL, lower limbs; g, Hedges’ g standardized mean difference.

The effect of the two treatments was significantly different in all MBUT sub-items. We found that the Ekso training led to a better improvement in the GSI and PSDI items as compared to the CG, with a moderate effect size (*g* = 0.56 and, respectively, *g* = 0.42) (Figure 2). 

This within- and between-group differences were still appreciable concerning the MBUT-LL (*g* = 0.42), but they did not achieve statistical significance. Even though this was not the primary aim in our study, we found some peculiar differences at both within- and between-group levels when considering AIS subgroups (grade A, i.e., a complete LM with no sensory or motor function in the sacral segments S4–5, or grade B, i.e., incomplete sensory LM, for which sensory function is preserved, but no motor function is present below the neurological level), as shown in Table 2 and Table 3.

### 3.2. Secondary Outcomes

**Figure 3:**
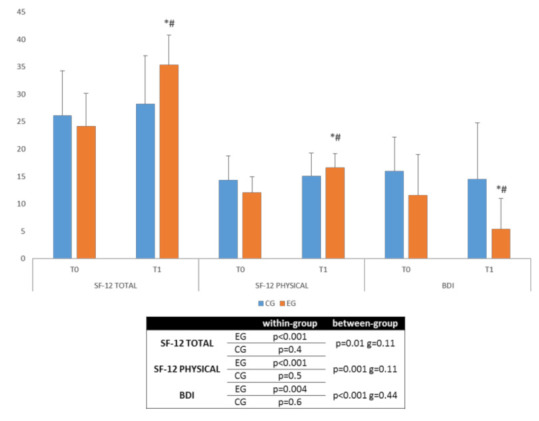
Secondary outcome measures (median + interquartile range). * significant within-group differences, # significant between-group differences. Legend: EG, experimental group; CG, control group; T0, evaluation at baseline; T1, evaluation at the end of the protocol; BDI, Beck Depression Inventory; SF-12 TOTAL, Short-Form-12 Health Survey Total; SF-12 PHYSICAL, Short-Form-12 Health Survey Physical; g, Hedges’ g standardized mean difference.

The Ekso training led to better results than CPT did in overall quality of life (SF-12 Total; *g* = 0.11) and in physical perception (SF-12 Physical; *g* = 0.11) as well as in mood (BDI; *g* = 0.44) but with low-to-moderate effect sizes (Figure 3).

## 4. Discussion

In recent years, many studies confirmed the beneficial effects of exoskeletons in motor rehabilitation of neurological diseases [47,52,53,57,58,59,60], including SCI [60,61,62,63]. In particular, it has been shown that this robotic training may increase gait and balance [60], favor the activation of various muscle districts [61], and reduce spasticity [62] in patients with SCI. In addition, other studies highlighted that exoskeleton gait training can improve physical activity parameters, increase walking time and energy expenditure, and affect body composition profile after SCI [63]. To the best of our knowledge, this is the first study evaluating the effectiveness of rehabilitative training with the robotic exoskeleton Ekso-GT in improving BR in patients with SCI. Indeed, the novelty of our study consisted in having specifically investigated the effect of such advanced training on BR. Although both treatments improved the patients’ outcomes, the Ekso training was associated with a moderate effect size in the following outcomes in comparison to CPT: improvement in BR, regarding the reduction of body discomfort, a partial improvement in the perception of legs and thighs, as well as quality of life. Only one other study investigated this important issue [52]. However, the study was carried out in patients with stroke receiving training with a stationary exoskeleton (i.e., the Lokomat, Hokoma, Switzerland) and evaluating the additional role of VR in further potentiating BR. More in detail, the Ekso-GT allowed patients with SCI to perform a nearly natural, independent walk, with an improvement of BR, which was probably related to the strong amount of multi-sensory information. Actually, a relevant finding emerged from the intra-group analysis of the EG with significant differences between patients with AIS-A (EG-A) and AIS-B (EG-B); i.e., EG-B had a higher BR recovery than EG-A, as demonstrated by the results of the BUT subscales. These data could be explained by the fact that EG-B had a greater perception of the body than EG-A owing to the sparing of the sensory functions. 

**Figure 4:**
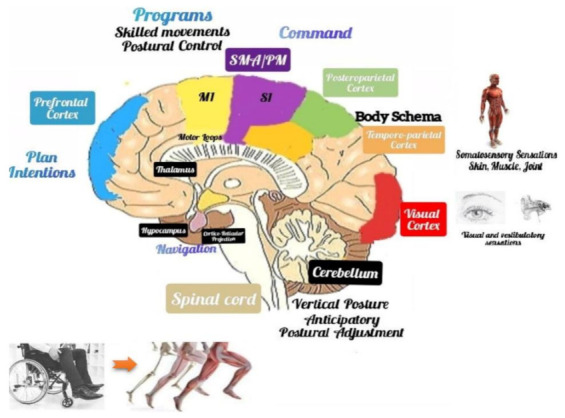
Represents the neural pathways that create the body representation, with signals from visual, vestibular/auditory, and somatosensory (proprioceptive) receptors flowing into the brain stem, cerebellum, thalamus, and cerebral cortex. Body representation (bottom figure) affects postural control and movement. The body scheme is constructed in the temporoparietal, vestibular, and posterior-parietal cortices, involving their connections with the cerebellum (top part of the figure). Legend: supplementary motor area (SMA) and the premotor area (PM).

In support of this issue, Scandola et al., in a virtual reality study on the representation of peripersonal space in patients with SCI, found that the patients who retained sensory functions reported benefits on motor learning if exposed to visual–motor feedback [64]. Besides, the same authors had previously stated that some cognitive functions, such as body, action, and space representations, are internalized and that somatosensory input and motor output might be necessary to create and keep a correct BR [65]. In fact, BR involves multiple brain areas, such as primary and motor somatosensory cortices, where body topographic maps are defined. Body maps adapt to the interactions with the physical environment [66]. The multisensory signals of the visual, vestibular, auditory, somatosensory (proprioceptive), and visceral receptors affect various areas of the central nervous system, allowing postural control and movement. These signals provide cognitive and emotional cues to the cerebral cortex and the limbic system, allowing purposeful voluntary movements or emotional motor behaviors. Voluntary movements result from intentional motor commands that involve the cerebral cortex, the brain stem, and the spinal cord. Instead, emotional motor behavior depends on projections from the limbic hypothalamus to the brainstem (i.e., fight or flight reactions) [67,68]. The cognitive process of controlling gait and posture requires a complex network (Figure 4).

The sensory signals from the skin, joint, and muscle receptors flow to the brain stem, cerebellum, thalamus, and cerebral cortex. At the cerebral cortex level, the signals from the visual, vestibular, and primary sensory cortex (S1) are integrated into an internal BR model, such as the body pattern and verticality, which is built in the temporal–parietal cortex, vestibular cortex, and posterior parietal cortex, where the body schema exists. The connection between the temporoparietal cortex and the cerebellum can contribute to this process. Thereafter, body information is transmitted to the supplementary motor area (SMA) and the premotor cortex (PMC) where this information can be used to elaborate the motor program. This information is also transferred to the hippocampus to explore other behaviors. The cortical motor areas, the basal ganglia, and the cerebellum build adequate motor programs. Finally, the body information generated in the vestibular cortex can be used to keep a vertical posture through the cortical–vestibular and vestibular–spinal tract. The signals of the prefrontal cortex, including plans and intentions, can trigger the execution of motor programs in the SMA/PMC, which may include intentional movements and postural control. The postural control program can be used to generate early postural adjustments through the cortico–reticular and reticulospinal tract. Then, the motor programs are sent to the M1, obtaining targeted and goal-oriented movements [68]. The interruption of afferent and/or efferent brain–body pathways promotes a wide cortical reorganization, with the homuncular reorganization of the sensorimotor areas and specific “somatotopic interference”. These neural interruptions, as occurs in SCI, create anomalies in the cortical representation of the body, where compensatory somatotopic remapping might occur [69].

In SCI, the sensorimotor signaling between the brain and the part of the body located below the level of the lesion is interrupted although the physical body remains unaffected [70,71]. The topographical reorganization of the body parts occurs in response to the activity and the acquisition of new skills [72]. In fact, both theoretical and quantitative studies [73,74,75] have shown that the acquisition of wheelchair competence by patients with SCI alters the metric perceptions of the body, suggesting an interaction between the body model and online information from the periphery updated through subsequent visual and proprioceptive changes in the position of the body. Therefore, it may be argued that the intense, repetitive, assisted, and task-oriented practice of gait and balance training using the Ekso-GT has a greater impact on BR changes than CPT does thanks to the multisensory stimulation-induced cortical neural plasticity. This is also confirmed by the fact that patients with spared sensibility had better BR improvements. This finding is intriguing given that BR is a modifiable factor through the different environmental stimulation, and its enhancement could have beneficial effects on the patient’s motor and psychological outcomes. In this regard, we formerly hypothesized that rehabilitation with robotic devices plus virtual reality feedback (i.e., an avatar that provides the patient with visual feedback of the movements performed and of the body) leads to an improvement in motor performance in patients with chronic hemiparesis [52]. Moreover, the recovery of BR together with the improvement of motor performance can increase the programming and execution of motor gestures, as BR and motor areas are strictly interconnected. Obviously, the success of rehabilitation training depends on individual internal and external factors, including motivation for treatment, mood, injury severity, pain, and comorbidities. Moreover, these factors may influence BR recovery. In fact, some studies have confirmed the relationship between BR improvement, injury severity, and secondary conditions [32]. 

Unfortunately, as Bailey et al. pointed out, BR is an often-overlooked aspect in patients with SCI [76]. In fact, other studies highlighted the importance of not underestimating BR in such patients in order to improve psychophysical well-being. In their study, van Diemen et al. suggested that during rehabilitation, patients affected by SCI experience body changes thanks to physical activities and sports, learning personal care, and performing other activities of daily life [37]. With this in mind, all rehabilitation team professionals, including psychologists, physiotherapists, and occupational therapists, can play a crucial role in improving the patient’s BR [37]. As confirmed by previous studies, our results show that the reconstruction of a BR can incentivize functional recovery, and this can have repercussions on normal daily activities and personal autonomy, which is another important issue in patients with SCI. Some authors found that patients who performed robotic training reported greater motor and psychological benefits [77,78]. To this end, an improvement in mood was also found in the EG (receiving robotic rehabilitation) as compared to the CG.

Juszczak et al. hypothesized that powered exoskeletons could lead to greater patient integration in the community and, consequently, a better quality of life [79]. We also found an improvement in the perception of quality of life with regard to physical health. Nonetheless, social integration was not assessed in our study, and this issue deserves investigation. In order to reach a better community integration, overground exoskeletons should be used as assistive and not only rehabilitative devices. 

The main limitations of the study are the small sample size and the absence of long-term follow-up assessment. Although the study was a priori powered, and 21 patients per arm were enough to demonstrate significant within- and between-group differences concerning the primary outcome, larger samples remain, however, mandatory to extend the results to the entire population of SCI patients. Furthermore, we did not consider other factors, including the influence of subjective factors such as perceived fatigue, pain, motor abilities, and other psychological constructs that could affect BR as well as the results of the training. Further studies using long-term follow-up should be promoted to investigate whether and to what extent the functional gain is maintained after the training. Finally, the BUT test used to evaluate body perception has not been validated in the SCI population although it has been largely used in other neurological patients. Furthermore, since only specific subscales or global measures were used, the real effectiveness of the subscales in evaluating a sample of SCI subjects should be taken into consideration.

## 5. Conclusions

In conclusion, SCI can change a patient’s BR, affecting his/her physical and psychological well-being. Based on our promising data, exoskeleton gait training could be useful for obtaining positive changes in BR, especially for the reduction of psychological distress and perception of legs/thighs, which both affect the clinical outcomes. This new approach may thus be a way to provide the patients with a more personalized rehabilitation. Even though other studies with larger samples and longer follow-up are needed to confirm these promising results, the use of new rehabilitation technologies could be a valuable way of promoting functional recovery of SCI patients not only to improve motor function but also to target BR.

## Figures and Tables

**Table 1:** Demographic and clinical characteristics of the patients. Mean ± standard deviation or numbers and percentages were used to describe the variables.

	Experimental Group	Control Group	All	*p*-Value
Patients	21	21	42	
Age (years)	58.6 ± 15.0	52.6 ± 9.0	55.6 ± 12.6	0.12
Gender				
Female	10 (40.0%)	7 (33.3%)	17 (40.5%)	0.35
Male	11 (60.0%)	14 (66.7%)	25 (59.5%)	
Education				
Elementary school	-	-	-	0.23
Middle school	5 (23.8%)	3 (14.3%)	8 (19.0%)	
High school	14 (66.7%)	12 (57.1%)	26 (62.0%)	
University	2 (9.5%)	6 (28.6%)	8 (19.0%)	
Spinal Injury Disability (AIS)				
AIS—A patients	10 (47.6%)	10 (47.6%)	20 (47.6%)	0.99
AIS—B patients	11 (52.3%)	11 (52.3%)	22 (52.3%)	
Time Post-Injury				
AIS—A patients	7 ± 1	6 ± 2	7 ± 2	0.93
AIS—B patients	6 ± 2	7 ± 2	7 ± 2	

**Table 2:** Statistical comparison of changes in the clinical score of a primary outcome measure of patients.

Clinical Scales	Group Analysis		Median (IQR)	*p*-Value
BUT-A (GSI)	T0–T0 between groups	CG A T0/EG A T0	0.74 (0.4)–1.1 (0.9)	0.60
		CG B T0/EG B T0	1.04 (0.4)–0.7 (0.5)	0.14
		CG A T0/CG B T0	0.74 (0.4)–1.04 (0.4)	0.12
		EG A T0/EG B T0	1.1 (0.9)–0.7 (0.5)	0.31
	T1–T1 between groups	CG A T1/EG A T1	0.62 (0.5)–0.45 (0.7)	0.49
		CG B T1/EG B T1	0.9 (0.5)–0.14 (0.3)	<0.001 *
		CG A T1/CG B T1	0.62 (0.5)–0.9 (0.5)	0.18
		EG A T1/EG B T1	0.45 (0.7)–0.14 (0.3)	0.18
	T0–T1 within group	CG A T0/CG A T1	0.74 (0.4)–0.62 (0.5)	0.09
		CG B T0/CG B T1	1.04 (0.4)–0.9 (0.5)	0.09
		EG A T0/EG A T1	1.1 (0.9)–0.45 (0.7)	<0.001 *
		EGB T0/EG B T1	0.7 (0.5)–0.14 (0.3)	<0.001 *
BUT-B (PSDI)	T0–T0 between groups	CG A T0/EG A T0	2.01 (0.5)–2.9 (1.7)	0.10
		CG B T0/EG B T0	3.03 (3.1)–3 (1.6)	0.95
		CG A T0/CG B T0	2.01 (0.5)–3.03 (3.1)	0.32
		EG A T0/EG B T0	2.9 (1.7)–3 (1.6)	0.91
	T1–T1 between groups	CG A T1/EG A T1	1.5 (0.5)–1.22 (1.3)	0.54
		CG B T1/EG B T1	2.7 (3.1)–1.3 (1.0)	0.17
		CG A T1/CG B T1	1.5 (0.5)–2.7 (3.1)	0.25
		EG A T1/EG B T1	1.22 (1.3)–1.3 (1.0)	0.88
	T0–T1 within group	CG A T0/CG A T1	2.01 (0.5)–1.5 (0.5)	0.01
		CG B T0/CG B T1	3.03 (3.1)–2.7 (3.1)	0.07
		EG A T0/EG A T1	2.9 (1.7)–1.22 (1.3)	0.01
		EGB T0/EG B T1	3 (1.6)–1.3 (1.0)	<0.001 *
BUT-B LL	T0–T0 between groups	CG A T0/EG A T0	2.9 (3.7)–3.6 (6.0)	0.76
		CG B T0/EG B T0	5.6 (3.7)–2.9 (2.7)	0.07
		CG A T0/CG B T0	2.9 (3.7)–5.6 (3.7)	0.11
		EG A T0/EG B T0	3.6 (6.0)–2.9 (2.7)	0.73
	T1–T1 between groups	CG A T1/EG A T1	3.1 (2.7)–1.3 (3.4)	0.21
		CG B T1/EG B T1	4.9 (3.0)–0.5 (1.0)	<0.001 *
		CG A T1/CG B T1	3.1 (2.7)–4.9 (3.0)	0.16
		EG A T1/EG B T1	1.3 (3.4)–0.5 (1.0)	0.49
	T0–T1 within group	CG A T0/CG A T1	2.9 (3.7)–3.1 (2.7)	0.68
		CG B T0/CG B T1	5.6 (3.7)–4.9 (3.0)	0.29
		EG A T0/EG A T1	3.6 (6.0)–1.3 (3.4)	0.05
		EGB T0/EG B T1	2.9 (2.7)–0.5 (1.0)	0.001 *

EG, experimental group; CG, control group; T0, evaluation at baseline; A, AIS A; B, AIS B; T1, evaluation at the end of the protocol; BUT-A, Body Uneasiness Test (part 1); BUT-B, Body Uneasiness Test (part 2); GSI, General Severity Index; PSDI, Positive Symptom Distress Index; LL, lower limbs; * statistical significance.

**Table 3:** Statistical comparison of changes in the clinical score of secondary outcome measures of patients.

Clinical Scales	Group Analysis		Median (IQR)	*p*-Value
SF-12 TOTAL	T0–T0 between groups	CG A T0/EG A T0	27.8 (8.1)–21.1 (5.7)	0.39
		CG B T0/EG B T0	24.5 (8.2)–26.9 (6.2)	0.45
		CG A T0/CG B T0	27.8 (8.1)–24.5 (8.2)	0.37
		EG A T0/EG B T0	21.1 (5.7)–26.9 (6.2)	0.49
	T1–T1 between groups	CG A T1/EG A T1	30.3 (10.2)–35 (4.5)	0.19
		CG B T1/EG B T1	26.1 (7.5)–35.7 (6.4)	<0.001 *
		CG A T1/CG B T1	30.3 (10.2)–26.1 (7.5)	0.29
		EG A T1/EG B T1	35 (4.5)–35.7 (6.4)	0.77
	T0–T1 within group	CG A T0/CG A T1	27.8 (8.1)–30.3 (10.2)	0.19
		CG B T0/CG B T1	24.5 (8.2)–26.1 (7.5)	0.03
		EG A T0/EG A T1	21.1 (5.7)–35 (4.5)	<0.001 *
		EG B T0/EG B T1	26.9 (6.2)–35.7 (6.4)	<0.001 *
SF-12 PHYSICAL	T0–T0 between groups	CG A T0/EG A T0	15.4 (5.3)–12.2 (2.4)	0.01
		CG B T0/EG B T0	13.3 (3.6)–11.9 (3.5)	0.38
		CG A T0/CG B T0	15.4 (5.3)–13.3 (3.6)	0.29
		EG A T0/EG B T0	12.2 (2.4)–11.9 (3.5)	0.83
	T1–T1 between groups	CG A T1/EG A T1	15.2 (4.7)–16.8 (2.6)	0.36
		CG B T1/EG B T1	15 (3.7)–16.5 (2.4)	0.29
		CG A T1/CG B T1	15.2 (4.7)–15 (3.7)	0.91
		EG A T1/EG B T1	16.8 (2.6)–16.5 (2.4)	0.75
	T0–T1 within group	CG A T0/CG A T1	15.4 (5.3)–15.2 (4.7)	0.90
		CG B T0/CG B T1	13.3 (3.6)–15 (3.7)	0.07
		EG A T0/EG A T1	12.2 (2.4)–16.8 (2.6)	<0.001 *
		EGB T0/EG B T1	11.9 (3.5)–16.5 (2.4)	<0.001 *
BDI	T0–T0 between groups	CG A T0/EG A T0	17.5 (6.3)–12.5 (7.3)	0.12
		CG B T0/EG B T0	14.5 (6.1)–107 (7.6)	0.21
		CG A T0/CG B T0	17.5 (6.3)–14.5 (6.1)	0.29
		EG A T0/EG B T0	15.5 (7.3)–10.7 (7.6)	0.59
	T1–T1 between groups	CG A T1/EG A T1	16.6 (4.3)–5.6 (6.3)	<0.001 *
		CG B T1/EG B T1	12.5 (6.2)–5.2 (4.8)	0.005
		CG A T1/CG B T1	16.6 (4.3)–12.5 (6.2)	0.09
		EG A T1/EG B T1	5.6 (6.3)–5.2 (4.8)	0.87
	T0–T1 within group	CG A T0/CG A T1	17.5 (6.3)–16.6 (14.3)	0.43
		CG B T0/CG B T1	14.5 (6.1)–12.5 (6.2)	<0.001 *
		EG A T0/EG A T1	12.5 (7.3)–5.6 (6.3)	<0.001 *
		EG B T0/EG B T1	10.7 (7.6)–5.2 (4.8)	0.14

EG, experimental group; CG, control group; T0, evaluation at baseline; A, AIS A; B, AIS B; T1, evaluation at the end of the protocol; BDI, Beck Depression Inventory; SF-12 TOTAL, Short-Form-12 Health Survey Total; SF-12 PHYSICAL, Short-Form-12 Health Survey Physical; * statistical significance.

## Data Availability

Data will be available on-demand to the corresponding author.
